# Benzoyl­dicarbon­yl(η^5^-inden­yl)ruthenium(II)

**DOI:** 10.1107/S1600536810033180

**Published:** 2010-08-25

**Authors:** Tracey A. Oudenhoven, Brennan J. Walder, James A. Golen, Arnold L. Rheingold, Jason S. D’Acchioli

**Affiliations:** a Department of Chemistry, The University of Wisconsin-Stevens Point, 2001 Fourth Avenue, Stevens Point, WI 54481, USA; b Department of Chemistry, The Ohio State University, 100 West 18th Avenue, Columbus, OH 43210, USA; c Department of Chemistry, UMass Dartmouth, 285 Old Westport Road, North Dartmouth, MA 02747, USA; d Department of Chemistry and Biochemistry, 9500 Gilman Drive, MC 0332, La Jolla, CA 92093, USA

## Abstract

In the title mol­ecule, [Ru(C_9_H_7_)(C_7_H_5_O)(CO)_2_], the dihedral angle between the mean plane of the indene ring system and the phenyl ring is 86.28 (8)°. The crystal structure is stabilized by weak inter­molecular C—H⋯O and C—H⋯π(arene) inter­actions. The Ru—η^5^-cyclopentadienyl centroid bond length is 1.946 (11) Å

## Related literature

For background information, see: Chung *et al.* (1982 ▶). For the synthetic procedure, see: Badger *et al.* (2009 ▶).
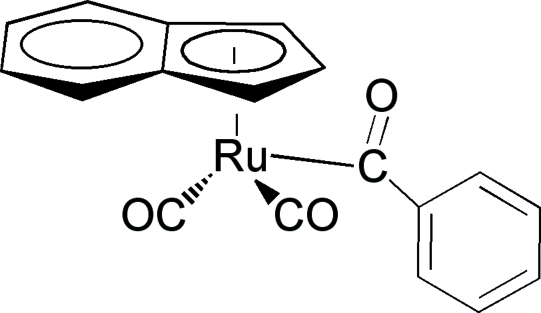


## Experimental

### 

#### Crystal data

[Ru(C_9_H_7_)(C_7_H_5_O)(CO)_2_]
*M* *_r_* = 377.35
Monoclinic, 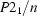

*a* = 11.531 (3) Å
*b* = 8.731 (2) Å
*c* = 15.158 (4) Å
β = 108.150 (3)°
*V* = 1450.2 (7) Å^3^
*Z* = 4
Mo *K*α radiation
μ = 1.09 mm^−1^
*T* = 100 K
0.30 × 0.10 × 0.10 mm

#### Data collection

Bruker APEXII CCD diffractometer
Absorption correction: multi-scan (*SADABS*; Bruker, 2008 ▶) *T*_min_ = 0.736, *T*_max_ = 0.899
11223 measured reflections
3332 independent reflections
2730 reflections with *I* > 2σ(*I*)
*R*_int_ = 0.037

#### Refinement

*R*[*F* ^2^ > 2σ(*F* ^2^)] = 0.033
*wR*(*F* ^2^) = 0.084
*S* = 1.05
3332 reflections
199 parameters
H-atom parameters constrained
Δρ_max_ = 1.07 e Å^−3^
Δρ_min_ = −0.63 e Å^−3^

### 

Data collection: *APEX2* (Bruker, 2008 ▶); cell refinement: *SAINT* (Bruker, 2008 ▶); data reduction: *SAINT*; program(s) used to solve structure: *SHELXS97* (Sheldrick, 2008 ▶); program(s) used to refine structure: *SHELXL97* (Sheldrick, 2008 ▶); molecular graphics: *Mercury* (Macrae *et al.*, 2006 ▶); software used to prepare material for publication: *SHELXTL* (Sheldrick, 2008 ▶).

## Supplementary Material

Crystal structure: contains datablocks I, global. DOI: 10.1107/S1600536810033180/lh5113sup1.cif

Structure factors: contains datablocks I. DOI: 10.1107/S1600536810033180/lh5113Isup2.hkl

Additional supplementary materials:  crystallographic information; 3D view; checkCIF report

## Figures and Tables

**Table 1:** Hydrogen-bond geometry (Å, °) *Cg* is the centroid of the C13–C18 ring.

*D*—H⋯*A*	*D*—H	H⋯*A*	*D*⋯*A*	*D*—H⋯*A*
C17—H17*A*⋯O3^i^	0.95	2.55	3.204 (4)	126
C9—H9*A*⋯*Cg*^ii^	0.95	2.72	3.657 (3)	169

Symmetry codes: (i) 
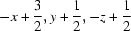
; (ii) 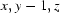.

**Fig. 1.**
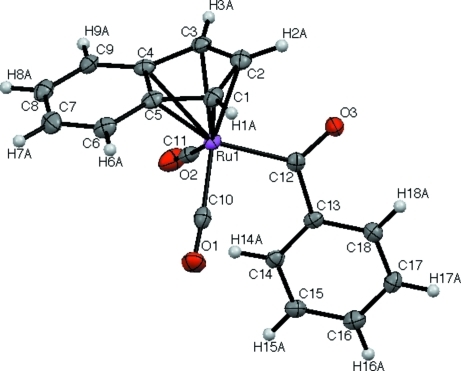
The molecular structure of the title compound. Displacement ellipsoids are drawn at the 50% probability level.

## supplementary crystallographic information

### Comment

The title compound was synthesized *via* attack of the Grignard reagent phenyl-magnesium bromide on [tricarbonyl(η^5^-indenyl)ruthenium(II)]^+^. It is well established that coordination of *ML*_n_ fragments (*M* = for example Fe, Ru, Rh; *L*_n_ = for example CO, phosphines) to an arene ring can result in nucleophilic or electrophilic attack at the arene ring or the carbonyl ligand (Chung *et al.*, 1982). The latter case is the subject of this structural study.

The molecular structure of the title compound is shown in Fig. 1. The dihedral angle between the mean plane of the indene ring system and the phenyl ring is 86.28 (8)°. The crystal structure is stabilized by weak intermolecular C-H···O and C-H···π(arene) interactions.

### Experimental

All reactions were performed under nitrogen using standard Schlenk techniques. THF was dried using a MBraun solvent purification system and used fresh. The compound benzoyldicarbonyl(η^5^-indenyl)ruthenium(II) was prepared by reaction of tricarbonyl(η^5^-indenyl)ruthenium(II) tetrafluoroborate with the Grignard reagent phenyl-magnesium bromide; tricarbonyl(η^5^-indenyl)ruthenium(II) tetrafluoroborate was prepared according to a previously established procedure (Badger *et al.*, 2009).

To a 100 ml three-neck round-bottom flask containing 15 ml of anhydrous THF at 273 K was added 142 mg tricarbonyl(η^6^-indenyl)ruthenium(II) tetrafluoroborate. Approximately 1.3 ml of 0.5 *M* phenyl-magnesium bromide (1.7 mol excess relative to tricarbonyl(η^5^-indenyl)ruthenium(II) tetrafluoroborate) was added to the flask. The solution changed from pale yellow to orange, and was allowed to stir for 10 minutes at 273 K. Excess Grignard reagent was killed with several drops of dilute HCl.

Solid magnesium salts precipitated out of the reaction mixture. The material was filtered *via* gravity filtration, and the solid was discarded. The mother liquor was extracted into diethyl ether and washed with two 10 ml aliquots of distilled water in a seperatory funnel. The organic layer was dried over MgSO_4_. The ether layer was removed *via* roto-evaporation, yielding a dark-yellow oil. X-ray diffraction-quality crystals precipitated from the oil on standing overnight.

### Refinement

All hydrogen atoms were placed in calculated positions with appropriate riding models. Carbon-bound H-atoms were placed in calculated positions (C—H 0.95 to 1.00 Å) with *U*(H) = 1.2 *U*(C).

### Crystal data

**Table:**

[Ru(C_9_H_7_)(C_7_H_5_O)(CO)_2_]	*F*(000) = 752
*M**_r_* = 377.35	*D*_x_ = 1.728 Mg m^−^^3^
Monoclinic, *P*2_1_/*n*	Mo *K*α radiation, λ = 0.71073 Å
Hall symbol: -P 2yn	Cell parameters from 5236 reflections
*a* = 11.531 (3) Å	θ = 2.7–28.1°
*b* = 8.731 (2) Å	µ = 1.09 mm^−^^1^
*c* = 15.158 (4) Å	*T* = 100 K
β = 108.150 (3)°	Block, yellow
*V* = 1450.2 (7) Å^3^	0.30 × 0.10 × 0.10 mm
*Z* = 4	

### Data collection

**Table:**

Bruker APEXII CCD diffractometer	3332 independent reflections
Radiation source: fine-focus sealed tube	2730 reflections with *I* > 2σ(*I*)
graphite	*R*_int_ = 0.037
φ and ω scans	θ_max_ = 28.1°, θ_min_ = 2.0°
Absorption correction: multi-scan (*SADABS*; Bruker, 2008)	*h* = −15→15
*T*_min_ = 0.736, *T*_max_ = 0.899	*k* = −11→11
11223 measured reflections	*l* = −19→13

### Refinement

**Table:**

Refinement on *F*^2^	Primary atom site location: structure-invariant direct methods
Least-squares matrix: full	Secondary atom site location: difference Fourier map
*R*[*F*^2^ > 2σ(*F*^2^)] = 0.033	Hydrogen site location: inferred from neighbouring sites
*wR*(*F*^2^) = 0.084	H-atom parameters constrained
*S* = 1.05	*w* = 1/[σ^2^(*F*_o_^2^) + (0.0372*P*)^2^ + 1.048*P*] where *P* = (*F*_o_^2^ + 2*F*_c_^2^)/3
3332 reflections	(Δ/σ)_max_ = 0.013
199 parameters	Δρ_max_ = 1.07 e Å^−^^3^
0 restraints	Δρ_min_ = −0.63 e Å^−^^3^

### Special details

**Table:**

Geometry. All e.s.d.'s (except the e.s.d. in the dihedral angle between two l.s. planes) are estimated using the full covariance matrix. The cell e.s.d.'s are taken into account individually in the estimation of e.s.d.'s in distances, angles and torsion angles; correlations between e.s.d.'s in cell parameters are only used when they are defined by crystal symmetry. An approximate (isotropic) treatment of cell e.s.d.'s is used for estimating e.s.d.'s involving l.s. planes.
Refinement. Refinement of *F*^2^ against ALL reflections. The weighted *R*-factor *wR* and goodness of fit *S* are based on *F*^2^, conventional *R*-factors *R* are based on *F*, with *F* set to zero for negative *F*^2^. The threshold expression of *F*^2^ > σ(*F*^2^) is used only for calculating *R*-factors(gt) *etc*. and is not relevant to the choice of reflections for refinement. *R*-factors based on *F*^2^ are statistically about twice as large as those based on *F*, and *R*- factors based on ALL data will be even larger.

### Fractional atomic coordinates and isotropic or equivalent isotropic displacement parameters (Å^2^)

**Table:**

	*x*	*y*	*z*	*U*_iso_*/*U*_eq_	
Ru1	0.44803 (2)	0.32604 (3)	0.139849 (16)	0.01609 (9)	
O1	0.2399 (2)	0.5526 (3)	0.07196 (17)	0.0285 (5)	
O2	0.4145 (2)	0.2330 (3)	0.32314 (17)	0.0328 (6)	
O3	0.67033 (18)	0.4987 (3)	0.20243 (16)	0.0239 (5)	
C1	0.4696 (3)	0.2829 (4)	0.0000 (2)	0.0214 (7)	
H1A	0.4618	0.3618	−0.0494	0.026*	
C2	0.5797 (3)	0.2380 (4)	0.0669 (2)	0.0229 (7)	
H2A	0.6621	0.2819	0.0736	0.027*	
C3	0.5550 (3)	0.1181 (4)	0.1223 (2)	0.0208 (6)	
H3A	0.6170	0.0622	0.1732	0.025*	
C4	0.4273 (3)	0.0737 (4)	0.0800 (2)	0.0193 (6)	
C5	0.3748 (3)	0.1751 (3)	0.0039 (2)	0.0203 (7)	
C6	0.2508 (3)	0.1615 (4)	−0.0498 (2)	0.0238 (7)	
H6A	0.2153	0.2294	−0.0999	0.029*	
C7	0.1830 (3)	0.0471 (4)	−0.0273 (2)	0.0270 (7)	
H7A	0.0996	0.0354	−0.0628	0.032*	
C8	0.2350 (3)	−0.0538 (4)	0.0478 (2)	0.0248 (7)	
H8A	0.1859	−0.1325	0.0609	0.030*	
C9	0.3538 (3)	−0.0408 (3)	0.1020 (2)	0.0216 (7)	
H9A	0.3866	−0.1073	0.1534	0.026*	
C10	0.3210 (3)	0.4708 (4)	0.1019 (2)	0.0216 (7)	
C11	0.4272 (3)	0.2735 (4)	0.2550 (2)	0.0205 (7)	
C12	0.5661 (3)	0.4974 (3)	0.2068 (2)	0.0179 (6)	
C13	0.5309 (3)	0.6283 (3)	0.2604 (2)	0.0170 (6)	
C14	0.4295 (3)	0.6266 (4)	0.2918 (2)	0.0184 (6)	
H14A	0.3732	0.5441	0.2752	0.022*	
C15	0.4098 (3)	0.7448 (4)	0.3472 (2)	0.0206 (7)	
H15A	0.3418	0.7409	0.3699	0.025*	
C16	0.4894 (3)	0.8680 (4)	0.3694 (2)	0.0234 (7)	
H16A	0.4760	0.9487	0.4071	0.028*	
C17	0.5889 (3)	0.8731 (4)	0.3362 (2)	0.0215 (7)	
H17A	0.6425	0.9587	0.3503	0.026*	
C18	0.6106 (3)	0.7542 (4)	0.2827 (2)	0.0190 (6)	
H18A	0.6795	0.7580	0.2611	0.023*	

### Atomic displacement parameters (Å^2^)

**Table:**

	*U*^11^	*U*^22^	*U*^33^	*U*^12^	*U*^13^	*U*^23^
Ru1	0.01864 (15)	0.01520 (14)	0.01457 (14)	0.00019 (10)	0.00536 (10)	−0.00036 (9)
O1	0.0257 (13)	0.0278 (13)	0.0293 (13)	0.0065 (11)	0.0045 (10)	0.0040 (11)
O2	0.0520 (16)	0.0240 (12)	0.0297 (14)	0.0046 (12)	0.0233 (13)	0.0050 (11)
O3	0.0189 (11)	0.0248 (12)	0.0284 (13)	−0.0022 (10)	0.0081 (10)	−0.0058 (10)
C1	0.0278 (17)	0.0195 (15)	0.0186 (16)	−0.0024 (14)	0.0096 (14)	−0.0029 (13)
C2	0.0268 (17)	0.0247 (17)	0.0201 (16)	−0.0011 (14)	0.0117 (14)	−0.0052 (14)
C3	0.0228 (16)	0.0174 (15)	0.0215 (16)	0.0029 (13)	0.0062 (13)	−0.0047 (13)
C4	0.0229 (16)	0.0197 (15)	0.0150 (15)	0.0035 (13)	0.0056 (12)	−0.0024 (12)
C5	0.0279 (17)	0.0197 (15)	0.0145 (15)	0.0020 (13)	0.0085 (13)	−0.0037 (12)
C6	0.0291 (18)	0.0242 (17)	0.0160 (16)	0.0021 (14)	0.0039 (14)	−0.0026 (13)
C7	0.0248 (17)	0.0313 (19)	0.0230 (17)	−0.0035 (15)	0.0048 (14)	−0.0115 (15)
C8	0.0328 (19)	0.0208 (16)	0.0238 (17)	−0.0047 (14)	0.0130 (15)	−0.0054 (14)
C9	0.0270 (17)	0.0160 (15)	0.0229 (16)	−0.0005 (13)	0.0092 (14)	−0.0028 (13)
C10	0.0242 (17)	0.0208 (15)	0.0198 (15)	−0.0061 (14)	0.0070 (13)	−0.0035 (13)
C11	0.0245 (17)	0.0141 (14)	0.0229 (17)	0.0037 (13)	0.0072 (14)	−0.0016 (13)
C12	0.0191 (15)	0.0181 (15)	0.0155 (14)	0.0005 (12)	0.0040 (12)	0.0030 (12)
C13	0.0195 (15)	0.0159 (14)	0.0134 (14)	0.0024 (12)	0.0019 (12)	0.0007 (12)
C14	0.0199 (15)	0.0160 (14)	0.0183 (15)	0.0015 (12)	0.0046 (12)	0.0021 (12)
C15	0.0229 (16)	0.0204 (16)	0.0204 (16)	0.0048 (13)	0.0092 (13)	0.0026 (13)
C16	0.0264 (17)	0.0217 (16)	0.0215 (16)	0.0035 (14)	0.0065 (14)	−0.0008 (13)
C17	0.0232 (16)	0.0194 (15)	0.0202 (16)	−0.0035 (13)	0.0042 (13)	−0.0014 (13)
C18	0.0184 (15)	0.0222 (16)	0.0161 (15)	0.0006 (13)	0.0049 (12)	0.0002 (13)

### Geometric parameters (Å, °)

**Table:**

Ru1—C10	1.884 (3)	C5—C6	1.413 (5)
Ru1—C11	1.891 (3)	C6—C7	1.376 (5)
Ru1—C12	2.064 (3)	C6—H6A	0.9500
Ru1—C1	2.243 (3)	C7—C8	1.416 (5)
Ru1—C3	2.257 (3)	C7—H7A	0.9500
Ru1—C2	2.272 (3)	C8—C9	1.365 (5)
Ru1—C4	2.367 (3)	C8—H8A	0.9500
Ru1—C5	2.369 (3)	C9—H9A	0.9500
O1—C10	1.152 (4)	C12—C13	1.528 (4)
O2—C11	1.144 (4)	C13—C14	1.393 (4)
O3—C12	1.225 (3)	C13—C18	1.405 (4)
C1—C2	1.412 (5)	C14—C15	1.394 (4)
C1—C5	1.458 (4)	C14—H14A	0.9500
C1—H1A	1.0000	C15—C16	1.386 (5)
C2—C3	1.425 (5)	C15—H15A	0.9500
C2—H2A	1.0000	C16—C17	1.389 (4)
C3—C4	1.464 (4)	C16—H16A	0.9500
C3—H3A	1.0000	C17—C18	1.387 (4)
C4—C9	1.416 (4)	C17—H17A	0.9500
C4—C5	1.431 (4)	C18—H18A	0.9500
			
C10—Ru1—C11	97.72 (13)	C9—C4—C3	132.7 (3)
C10—Ru1—C12	89.69 (13)	C5—C4—C3	107.7 (3)
C11—Ru1—C12	88.61 (13)	C9—C4—Ru1	124.3 (2)
C10—Ru1—C1	97.41 (13)	C5—C4—Ru1	72.53 (17)
C11—Ru1—C1	156.27 (13)	C3—C4—Ru1	67.50 (16)
C12—Ru1—C1	109.65 (11)	C6—C5—C4	120.7 (3)
C10—Ru1—C3	154.88 (13)	C6—C5—C1	131.9 (3)
C11—Ru1—C3	98.33 (12)	C4—C5—C1	107.4 (3)
C12—Ru1—C3	109.88 (12)	C6—C5—Ru1	124.7 (2)
C1—Ru1—C3	61.82 (12)	C4—C5—Ru1	72.31 (18)
C10—Ru1—C2	130.72 (13)	C1—C5—Ru1	66.91 (16)
C11—Ru1—C2	131.54 (13)	C7—C6—C5	118.0 (3)
C12—Ru1—C2	92.65 (12)	C7—C6—H6A	121.0
C1—Ru1—C2	36.43 (12)	C5—C6—H6A	121.0
C3—Ru1—C2	36.67 (12)	C6—C7—C8	121.3 (3)
C10—Ru1—C4	122.10 (12)	C6—C7—H7A	119.4
C11—Ru1—C4	95.82 (12)	C8—C7—H7A	119.4
C12—Ru1—C4	146.71 (11)	C9—C8—C7	121.8 (3)
C1—Ru1—C4	60.58 (11)	C9—C8—H8A	119.1
C3—Ru1—C4	36.83 (11)	C7—C8—H8A	119.1
C2—Ru1—C4	60.11 (11)	C8—C9—C4	118.6 (3)
C10—Ru1—C5	94.31 (12)	C8—C9—H9A	120.7
C11—Ru1—C5	123.70 (12)	C4—C9—H9A	120.7
C12—Ru1—C5	146.36 (11)	O1—C10—Ru1	174.3 (3)
C1—Ru1—C5	36.72 (11)	O2—C11—Ru1	176.0 (3)
C3—Ru1—C5	60.65 (11)	O3—C12—C13	116.7 (3)
C2—Ru1—C5	59.98 (11)	O3—C12—Ru1	119.2 (2)
C4—Ru1—C5	35.16 (10)	C13—C12—Ru1	124.1 (2)
C2—C1—C5	108.0 (3)	C14—C13—C18	118.8 (3)
C2—C1—Ru1	72.91 (18)	C14—C13—C12	124.3 (3)
C5—C1—Ru1	76.37 (17)	C18—C13—C12	116.8 (3)
C2—C1—H1A	125.5	C13—C14—C15	120.7 (3)
C5—C1—H1A	125.5	C13—C14—H14A	119.7
Ru1—C1—H1A	125.5	C15—C14—H14A	119.7
C1—C2—C3	109.1 (3)	C16—C15—C14	120.1 (3)
C1—C2—Ru1	70.65 (17)	C16—C15—H15A	120.0
C3—C2—Ru1	71.08 (17)	C14—C15—H15A	120.0
C1—C2—H2A	125.4	C15—C16—C17	119.8 (3)
C3—C2—H2A	125.4	C15—C16—H16A	120.1
Ru1—C2—H2A	125.4	C17—C16—H16A	120.1
C2—C3—C4	107.1 (3)	C18—C17—C16	120.5 (3)
C2—C3—Ru1	72.25 (18)	C18—C17—H17A	119.8
C4—C3—Ru1	75.67 (17)	C16—C17—H17A	119.8
C2—C3—H3A	126.0	C17—C18—C13	120.2 (3)
C4—C3—H3A	126.0	C17—C18—H18A	119.9
Ru1—C3—H3A	126.0	C13—C18—H18A	119.9
C9—C4—C5	119.6 (3)		
			
C10—Ru1—C1—C2	158.7 (2)	C2—C1—C5—C6	−176.7 (3)
C11—Ru1—C1—C2	−72.1 (4)	Ru1—C1—C5—C6	116.7 (3)
C12—Ru1—C1—C2	66.4 (2)	C2—C1—C5—C4	5.3 (3)
C3—Ru1—C1—C2	−36.26 (19)	Ru1—C1—C5—C4	−61.4 (2)
C4—Ru1—C1—C2	−78.5 (2)	C2—C1—C5—Ru1	66.6 (2)
C5—Ru1—C1—C2	−114.0 (3)	C10—Ru1—C5—C6	−29.4 (3)
C10—Ru1—C1—C5	−87.27 (19)	C11—Ru1—C5—C6	72.8 (3)
C11—Ru1—C1—C5	41.9 (4)	C12—Ru1—C5—C6	−125.4 (3)
C12—Ru1—C1—C5	−179.61 (18)	C1—Ru1—C5—C6	−126.0 (4)
C3—Ru1—C1—C5	77.78 (19)	C3—Ru1—C5—C6	152.7 (3)
C2—Ru1—C1—C5	114.0 (3)	C2—Ru1—C5—C6	−164.8 (3)
C4—Ru1—C1—C5	35.54 (17)	C4—Ru1—C5—C6	115.5 (3)
C5—C1—C2—C3	−8.0 (3)	C10—Ru1—C5—C4	−144.92 (18)
Ru1—C1—C2—C3	60.9 (2)	C11—Ru1—C5—C4	−42.7 (2)
C5—C1—C2—Ru1	−69.0 (2)	C12—Ru1—C5—C4	119.1 (2)
C10—Ru1—C2—C1	−28.4 (3)	C1—Ru1—C5—C4	118.5 (3)
C11—Ru1—C2—C1	149.2 (2)	C3—Ru1—C5—C4	37.23 (17)
C12—Ru1—C2—C1	−120.3 (2)	C2—Ru1—C5—C4	79.68 (19)
C3—Ru1—C2—C1	119.2 (3)	C10—Ru1—C5—C1	96.6 (2)
C4—Ru1—C2—C1	79.9 (2)	C11—Ru1—C5—C1	−161.14 (19)
C5—Ru1—C2—C1	39.09 (18)	C12—Ru1—C5—C1	0.7 (3)
C10—Ru1—C2—C3	−147.6 (2)	C3—Ru1—C5—C1	−81.2 (2)
C11—Ru1—C2—C3	30.0 (3)	C2—Ru1—C5—C1	−38.78 (19)
C12—Ru1—C2—C3	120.5 (2)	C4—Ru1—C5—C1	−118.5 (3)
C1—Ru1—C2—C3	−119.2 (3)	C4—C5—C6—C7	−0.8 (4)
C4—Ru1—C2—C3	−39.30 (18)	C1—C5—C6—C7	−178.7 (3)
C5—Ru1—C2—C3	−80.1 (2)	Ru1—C5—C6—C7	−89.7 (3)
C1—C2—C3—C4	7.6 (3)	C5—C6—C7—C8	0.5 (5)
Ru1—C2—C3—C4	68.2 (2)	C6—C7—C8—C9	1.0 (5)
C1—C2—C3—Ru1	−60.7 (2)	C7—C8—C9—C4	−2.1 (4)
C10—Ru1—C3—C2	73.1 (3)	C5—C4—C9—C8	1.8 (4)
C11—Ru1—C3—C2	−157.7 (2)	C3—C4—C9—C8	180.0 (3)
C12—Ru1—C3—C2	−66.2 (2)	Ru1—C4—C9—C8	90.1 (3)
C1—Ru1—C3—C2	36.03 (19)	C11—Ru1—C10—O1	−128 (3)
C4—Ru1—C3—C2	113.7 (3)	C12—Ru1—C10—O1	143 (3)
C5—Ru1—C3—C2	78.1 (2)	C1—Ru1—C10—O1	34 (3)
C10—Ru1—C3—C4	−40.6 (4)	C3—Ru1—C10—O1	1(3)
C11—Ru1—C3—C4	88.60 (19)	C2—Ru1—C10—O1	50 (3)
C12—Ru1—C3—C4	−179.86 (17)	C4—Ru1—C10—O1	−26 (3)
C1—Ru1—C3—C4	−77.63 (19)	C5—Ru1—C10—O1	−3(3)
C2—Ru1—C3—C4	−113.7 (3)	C10—Ru1—C11—O2	128 (4)
C5—Ru1—C3—C4	−35.53 (16)	C12—Ru1—C11—O2	−142 (4)
C2—C3—C4—C9	177.5 (3)	C1—Ru1—C11—O2	−1(4)
Ru1—C3—C4—C9	−116.6 (3)	C3—Ru1—C11—O2	−32 (4)
C2—C3—C4—C5	−4.2 (3)	C2—Ru1—C11—O2	−50 (4)
Ru1—C3—C4—C5	61.7 (2)	C4—Ru1—C11—O2	5(4)
C2—C3—C4—Ru1	−65.9 (2)	C5—Ru1—C11—O2	28 (4)
C10—Ru1—C4—C9	−71.8 (3)	C10—Ru1—C12—O3	−139.5 (3)
C11—Ru1—C4—C9	31.1 (3)	C11—Ru1—C12—O3	122.8 (3)
C12—Ru1—C4—C9	127.5 (3)	C1—Ru1—C12—O3	−41.7 (3)
C1—Ru1—C4—C9	−151.4 (3)	C3—Ru1—C12—O3	24.5 (3)
C3—Ru1—C4—C9	127.3 (3)	C2—Ru1—C12—O3	−8.7 (3)
C2—Ru1—C4—C9	166.4 (3)	C4—Ru1—C12—O3	24.3 (4)
C5—Ru1—C4—C9	−114.3 (3)	C5—Ru1—C12—O3	−42.1 (4)
C10—Ru1—C4—C5	42.6 (2)	C10—Ru1—C12—C13	38.8 (2)
C11—Ru1—C4—C5	145.47 (19)	C11—Ru1—C12—C13	−59.0 (2)
C12—Ru1—C4—C5	−118.2 (2)	C1—Ru1—C12—C13	136.5 (2)
C1—Ru1—C4—C5	−37.12 (18)	C3—Ru1—C12—C13	−157.3 (2)
C3—Ru1—C4—C5	−118.4 (2)	C2—Ru1—C12—C13	169.5 (2)
C2—Ru1—C4—C5	−79.28 (19)	C4—Ru1—C12—C13	−157.5 (2)
C10—Ru1—C4—C3	160.97 (18)	C5—Ru1—C12—C13	136.1 (2)
C11—Ru1—C4—C3	−96.13 (19)	O3—C12—C13—C14	−163.6 (3)
C12—Ru1—C4—C3	0.2 (3)	Ru1—C12—C13—C14	18.1 (4)
C1—Ru1—C4—C3	81.28 (19)	O3—C12—C13—C18	13.1 (4)
C2—Ru1—C4—C3	39.12 (18)	Ru1—C12—C13—C18	−165.1 (2)
C5—Ru1—C4—C3	118.4 (2)	C18—C13—C14—C15	−2.4 (5)
C9—C4—C5—C6	−0.4 (4)	C12—C13—C14—C15	174.3 (3)
C3—C4—C5—C6	−179.0 (3)	C13—C14—C15—C16	2.0 (5)
Ru1—C4—C5—C6	−120.4 (3)	C14—C15—C16—C17	−0.1 (5)
C9—C4—C5—C1	178.0 (3)	C15—C16—C17—C18	−1.4 (5)
C3—C4—C5—C1	−0.6 (3)	C16—C17—C18—C13	0.9 (5)
Ru1—C4—C5—C1	57.92 (19)	C14—C13—C18—C17	0.9 (5)
C9—C4—C5—Ru1	120.0 (3)	C12—C13—C18—C17	−176.0 (3)
C3—C4—C5—Ru1	−58.55 (19)		

### Hydrogen-bond geometry (Å, °)

**Table:**

Cg is the centroid of the C13–C18 ring.

**Table:**

*D*—H···*A*	*D*—H	H···*A*	*D*···*A*	*D*—H···*A*
C17—H17A···O3^i^	0.95	2.55	3.204 (4)	126
C9—H9A···Cg^ii^	0.95	2.72	3.657 (3)	169

Symmetry codes: (i) −*x*+3/2, *y*+1/2, −*z*+1/2; (ii) *x*, *y*−1, *z*.
